# Evidence for family-level variation of phenotypic traits in response to temperature of Brazilian *Nyssorhynchus darlingi*

**DOI:** 10.1186/s13071-020-3924-7

**Published:** 2020-02-10

**Authors:** Virginia M. Chu, Maria Anice Mureb Sallum, Timothy E. Moore, Kevin J. Emerson, Carl D. Schlichting, Jan E. Conn

**Affiliations:** 1 Department of Biomedical Sciences, School of Public Health, State University of New York at Albany, 150 New Scotland Avenue, Albany, NY USA; 2 Wadsworth Center, New York State Department of Health, New York State Route 5, Albany, NY USA; 3 Faculdade de Saúde Pública, Universidade de São Paulo, São Paulo, SP Brazil; 4 Department of Ecology and Evolutionary Biology, University of Connecticut, Hartford, CT USA; 5 Biology Department, St. Mary’s College of Maryland, St. Mary’s City, Maryland USA

**Keywords:** *Nyssorhynchus darlingi*, Population structure, Single nucleotide polymorphisms (SNPs), Life history traits, Common garden experiment

## Abstract

**Background:**

*Nyssorhynchus darlingi* (also known as *Anopheles darlingi*) is the primary malaria vector in the Amazon River Basin. In Brazil, analysis of single nucleotide polymorphisms (SNPs) previously detected three major population clusters, and a common garden experiment in a laboratory setting revealed significant population variation in life history traits. Increasing temperatures and local level variation can affect life history traits, i.e. adult longevity, that alter vectorial capacity with implications for malaria transmission in *Ny. darlingi*.

**Methods:**

We investigated the population structure of *Ny. darlingi* from 7 localities across Brazil utilizing SNPs and compared them to a comprehensive *Ny. darlingi* catalog. To test the effects of local level variation on life history traits, we reared F_1_ progeny from the 7 localities at three constant temperatures (20, 24 and 28 °C), measuring key life history traits (larval development, food-starved adult lifespan, adult size and daily survival).

**Results:**

Using nextRAD genotyping-by-sequencing, 93 of the field-collected *Ny. darlingi* were genotyped at 33,759 loci. Results revealed three populations (*K* = 3), congruent with major biomes (Amazonia, Cerrado and Mata Atlântica), with greater *F*_ST_ values between biomes than within. In the life history experiments, increasing temperature reduced larval development time, adult lifespan, and wing length in all localities. The variation of family responses for all traits within four localities of the Amazonia biome was significant (ANOVA, *P* < 0.05). Individual families within localities revealed a range of responses as temperature increased, for larval development, adult lifespan, wing length and survival time.

**Conclusions:**

SNP analysis of several Brazilian localities provided results in support of a previous study wherein populations of *Ny. darlingi* were clustered by three major Brazilian biomes. Our laboratory results of temperature effects demonstrated that population variation in life history traits of *Ny. darlingi* exists at the local level, supporting previous research demonstrating the high plasticity of this species. Understanding this plasticity and inherent variation between families of *Ny. darlingi* at the local level should be considered when deploying intervention strategies and may improve the likelihood of successful malaria elimination in South America.
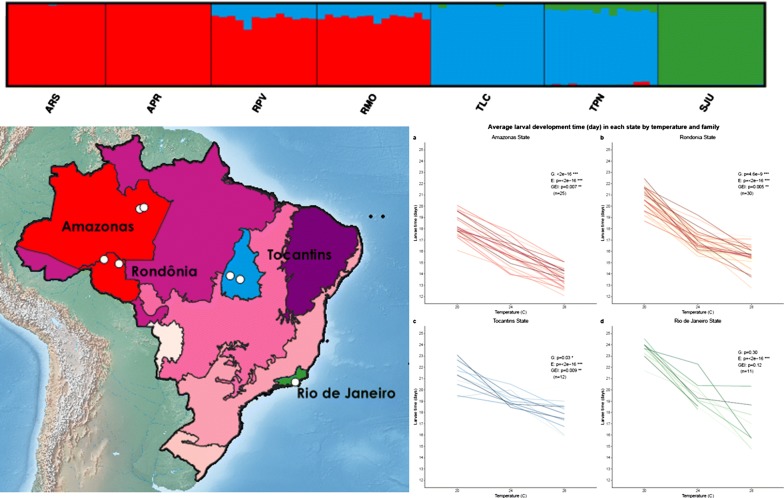

## Background

Malaria has made a comeback in Latin America in the last few years despite a recent period of decline from 2000 to 2014 [1, 2]. The Americas is the only region to have an increase in malaria mortality in 2017 compared to 2010, with a greater number of malaria cases reported in Venezuela, Brazil, and Nicaragua during this period [2]. The main vector and driver of this disease in South America is *Nysorrhynchus darlingi* (also known as *Anopheles darlingi* [3]), that exhibits significant geographical variation in behavior [4, 5] and in phenotypic plasticity [6–8]. This species has a natural infection rate by *Plasmodium* of up to 20% [5, 9], though a more common rate is 1–5% [10–12]. Adult vector traits relevant to disease transmission, such as adult life span and body size [13], can vary between populations [6, 7] and are influenced by larval development conditions such as food quantity [14] and temperature [15]. Globally, temperatures are projected to rise between 1–4 °C due to climate change [16]. Whereas even small changes in temperature may reduce vectorial capacity [17], the effects of temperature are not uniform across *Ny. darlingi* populations [7]. To be successful, future interventions in this region require a better understanding of this vector in a changing environment. Here we assess levels of genetic and phenotypic differentiation among *Ny. darlingi* populations from Brazil.

The geographical distribution of *Nyssorhynchus darlingi* includes diverse South American biomes [18] and is associated with a range of larval habitat types, including natural breeding sites with clean, shaded water and aquatic vegetation near human dwellings [19], as well as anthropogenic habitats, such as fish ponds [20] and dams [21]. Habitat modification, e.g. deforestation, was linked to *Ny. darlingi* presence in Peruvian breeding habitats [22] and has been positively correlated with malaria cases in Brazil [23]. A mathematical model using field-collected data found that the high biting rate and susceptibility to *Plasmodium* of *Ny. darlingi* in the Brazilian Amazon led to a high basic reproductive rate (*R*_*0*_) of malaria (mainly caused by *Plasmodium vivax*) [24]. The heterogeneity in distribution, vector competence and vectorial capacity of *Ny. darlingi* presents a major challenge to malaria elimination.

Research on the effects of juvenile stages on adult traits has increased the understanding of developmental trade-offs. Changes in life history traits, such as body size and adult survival, can modify vectorial capacity [25] and directly impact malaria transmission. In a theoretical climate risk model, the inclusion of the effects of temperature during the full life-cycle, such as juvenile development rate and mortality, revealed that mosquito populations are more sensitive to changes in temperature than adult data alone would indicate [26]. A study of full-sib F_1_ progeny from field collected *An. coluzzii* (formerly known as *An. gambiae* M form [27], Burkina Faso) found adult longevity increased with adult body size but decreased with longer larval development [28]. Population differentiation for both larval and adult life history traits of *Ny. darlingi* has been reported at the regional level in Brazil [6, 7], but variation at smaller scales is unstudied.

Average global temperatures are projected to increase 1–4 °C over the next 100 years because of climate change [16] and tropical insect populations are anticipated to be more negatively affected compared to those in temperate regions [29]. Exotherm development is very sensitive to temperature, which can affect traits relevant to disease transmission, such as body size and adult fitness [15, 25]. Laboratory rearing of *An. gambiae* suggested an upper thermal limit of 31 °C and complete larvae mortality at 35 °C, with increasing temperatures reducing adult body size and egg production [30, 31]. A malaria model predicted optimal transmission at 25 °C and was validated by an independent malaria transmission data set for *An. gambiae* (*s.l*.) and *P. falciparum* [32]. Modeled parasite and mosquito development rate reached a peak at 30 °C, in contrast, vector competence and vector survivorship peaked at 25 °C.

The analysis of single nucleotide polymorphisms (SNPs) in *Anopheles* has shed light on population structure [33, 34] and phenotypes [35, 36]. However, results of tests using SNP data to identify population structure of *Ny. darlingi* in South America have been mixed. Analysis of *Ny. darlingi* collected from 12 states across Brazil detected three genetic clusters [37] associated with major biogeographical regions. In contrast, analysis of specimens from three sites within a single biome (Amazonia), between 60–700 km apart, detected significant population divergence at a regional scale [38], although a subsequent analysis of two of these sites (60 km apart, new *vs* old settlement) to test for local differentiation in biting behavior found no significant genetic variation [4]. Despite similar methods used in these studies, comparisons between datasets are difficult given the variation in identified loci.

The aim of this study was to investigate local level variation in population structure and in life history traits of *Ny. darlingi* using a common garden experiment approach to address the following questions: (i) What is the scale of genetic differentiation among populations of *Ny darlingi*? and (ii) Is there evidence of small-scale variation in life history traits and in plastic responses to temperature variation? Our research combined broad-scale population genetic assays with empirical data from a common garden experiment. We investigated the effects of variation in rearing temperature on a major Neotropical vector, allowing us to assess the extent to which population differences in life history traits were due to environment (temperature), genetics, or both.

Here, we identified molecular genetic variation across biomes, significant phenotypic and genetic variation in life history traits, as well as within-population genetic variation for plasticity of *Ny. darlingi*. This variation could help tailor current intervention efforts, such as long-lasting insecticide nets (LLINs), indoor residual spraying (IRS) and larval source management (LSM), to regional and local scales for maximum efficiency and malaria elimination.

## Methods

In this study, we first analyzed the population structure of Brazilian *Ny. darlingi* with mosquitoes collected within the same year, over a range of seven locality sites. In order to increase our chances of identifying fine-scale population genetic structure, we created a catalog incorporating sequences of *Ny. darlingi* from Peru and Brazil and used this to reexamine the population structure of Brazilian *Ny. darlingi* and to investigate the possibility of fine-scale differentiation within three Brazilian biomes. We then investigated the among- and within-locality variation in life history traits such as larval development time, adult lifespan and body size, among these seven localities across Brazil. A common garden experiment of the mosquito populations from the seven localities was conducted in three constant temperature environmental chambers. Mosquitoes were observed from egg hatch to adult death and life history traits recorded.

### Study area and field collections

**Fig. 1:**
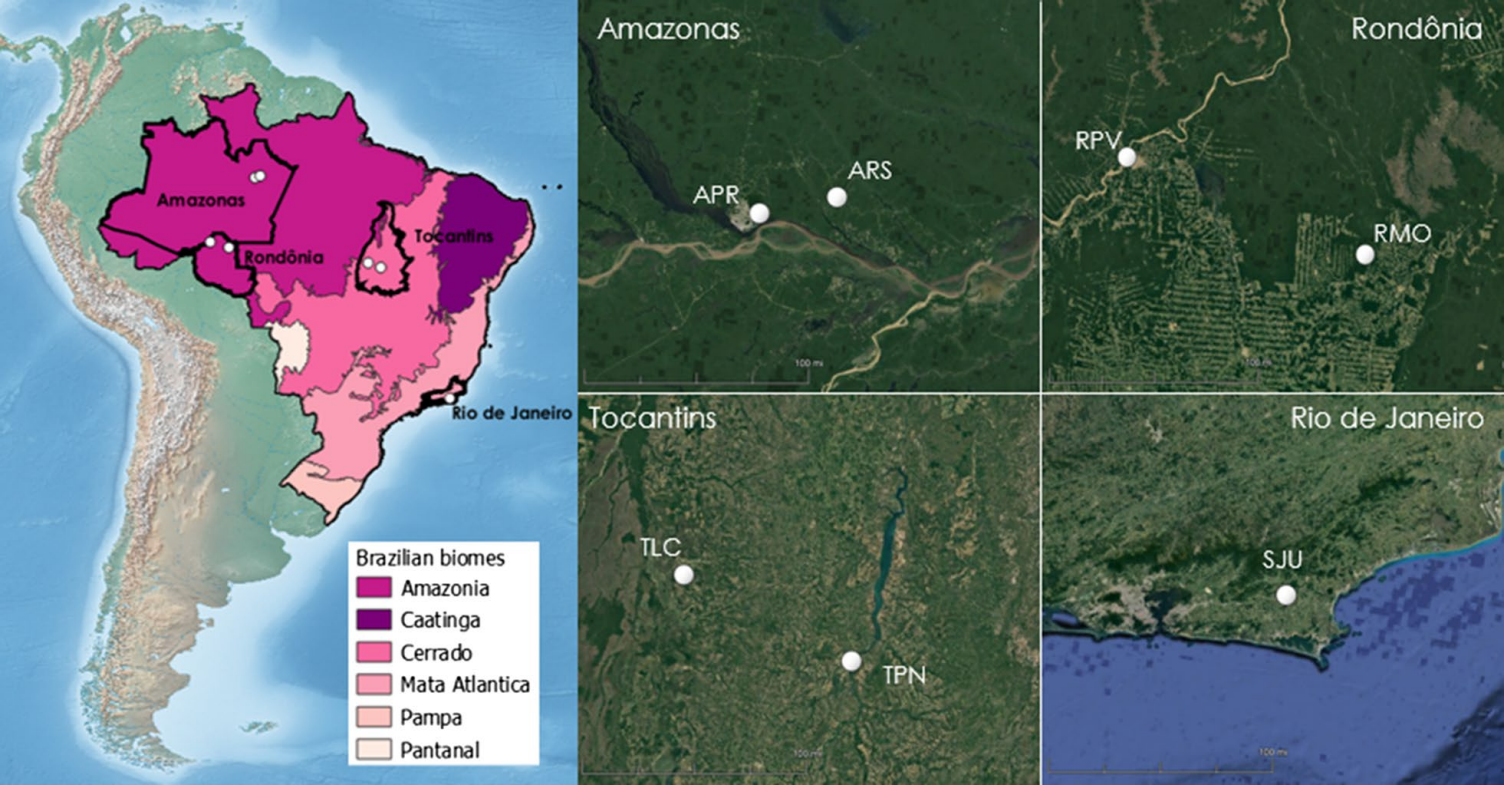
Map (with topography) of collection sites. Inset maps by state *via* GoogleEarth [72]

Adult female *Ny. darlingi* mosquitoes were collected from 7 localities across Brazil (Table 1, Fig. 1), spanning four states and 3 biomes. Details of the collection site criteria for paired sites are found in [7]. Mosquitoes were collected in the evening for 5 hours (17:00–22:00 h) using barrier screens as described in Moreno et al. [12] for 1–5 days, depending on locality and successful collection of the target species, *Ny. darlingi*. Blood-fed female mosquitoes from barrier screens were morphologically identified as *Ny. darlingi* [39] and maintained individually in a humid box and provided *ad libitum* sucrose solution during transport to the laboratory in São Paulo, Brazil (Laboratório de Entomologia de Saúde Pública – Culicidae, Faculdade de Saúde Pública, Universidade de São Paulo).

**Table 1:** Collection site information and *Ny. darlingi* details

Biome	State	Locality (abbreviation)	Month	Latitude	Avg. yearly T (°C)	No. collected	No. sequenced for SNPs	No. analyzed for life history
Amazonia	Amazonas	Ramal Novo Horizonte (ARS)	October	− 2.864	27.61	20	12	10
		Manaus-Brasilierinho (APR)	October	− 3.028	27.71	53	13	15
	Rondônia	Porto Velho (RPV)	July	− 8.742	25.70	95	13	15
		Machadinho D’Oeste (RMO)	July	− 9.223	25.60	44	14	15
Cerrado	Tocantins	Lagoa da Confusão (TLC)	March	− 10.7	27.35	34	14	8
		Porto Nacional (TPN)	March	− 10.796	27.54	19	14	4
Mata Atlântica	Rio de Janeiro	Lake Juturnaiba (SJU)	May	− 22.611	21.20	13	13	11

*Notes*: No. collected and No. sequenced for SNPs are individual female *Ny. darlingi*; No. analyzed for life history are the number of families from individual females. All localities were visited in 2016. Temperature data were obtained from online data sources (INMET [66] and SEDAM [67]]). Additional temperature data in Additional file 1: Table S3

*Abbreviations*: Avg, average; T, temperature

### Laboratory rearing

The laboratory rearing for progeny of field-caught individuals was carried out as previously described [7]. Briefly, eggs laid by individual females from each locality, referred to as families, were allowed to hatch and larvae were equally divided (*n* = 15) into each of three constant temperature environmental chambers (20, 24 and 28 ± 1 °C) (Additional file 1: Table S1) with a 12:12 h light:dark cycle and a relative humidity of 70–80%. Larvae were fed *ad libitum* and water was changed every other day; adults were provided only water after emergence. Larval, pupal and adult development was assessed daily. Mosquito specimens were maintained in these chambers until natural adult death, whereupon the left wing was collected for body size estimation.

### DNA extraction and nextRAD preparation

A subset (*n* = 93) of the field-collected *Ny. darlingi* (used to create the families in the life history experiment) was genotyped to evaluate population structure (Table 1). Individuals were selected from each of the 7 localities (*n* = 12–14 per locality) based on (i) successful egg laying (with priority given to the dams of the families used in the life history research); (ii) complete wing data; and (iii) a DNA concentration between 2.87 and 16.2 mg/ml. Genomic DNA was extracted from all specimens using Qiagen DNeasy Blood and Tissue kit (Qiagen, Germantown, MD, USA) and concentrations were quantified with a Qubit Fluorometer (Thermo Fisher Scientific, Waltham, MA, USA). Individuals were sequenced using nextRAD genotyping-by-sequencing methods as described in Emerson et al. [37] (SNPSaurus, LLC, Eugene, OR, USA). Briefly, the genomic DNA was first fragmented using a Nextera reaction to ligate adapter sequences to the fragments. The fragments were then amplified with an 8 bp Nextera primer (5′-TGC AGG AG-3′), and the library was pooled and purified, with size selection between 350–500 bp. The resulting library was then sequenced, generating 150 bp reads on two lanes of an Illumina HiSeq 4000.

### Data analysis

#### Nyssorhynchus darlingi catalog creation

All raw sequences were analyzed using STACKS v2.3b [40]. Sequences of 24 representative field collected female *Ny. darlingi* were used to create a catalog using STACKS *cstacks*, permitting 4 mismatches between stacks and enabling gapped alignments. In order to generalize the catalog to be useful across projects, samples used in this catalog were from previous publications [37, 41, 42]; similar sequencing methods were employed. This catalog consisted of 13 individuals from this study (the life history localities) and additional *Ny. darlingi* collected between 2006–2016 from Brazil (*n* = 7, additional states: Pará, São Paulo, Acre, Espirito Santo, Mato Grosso [37]) and Peru (*n* = 4, Lupuna and Cahuide, Loreto Department [41]). The *process radtags* program was used to drop low quality sequence reads and *ustacks* aligned reads into stacks with the following parameters: minimum depth of coverage for stack creation was set to 3, maximum distance allowed between stacks set to 4, and the maximum distance allowed to align secondary reads to primary reads set to 6. This generated a master catalog from *Ny. darlingi* sequences using consistent loci which allows for parallels to be drawn from different research projects.

#### nextRAD data analysis

For the present study, the sequences of the 93 *Ny. darlingi* from the seven collection localities (Table 1) were processed with *process radtags* and *ustacks* program as described above, compared against the above described catalog, and then SNPs were called with the STACKS -*sstacks*, -*tsv2bam* and -*gstacks* programsʼ settings set to default. The STACKS *populations* program was used to select a single SNP from each locus found in at least 40% of individuals in the dataset, a threshold slightly more lenient than the 50% used in previous population structure research [37]; this modification resulted in a greater number of loci for comparison.

STRUCTURE analysis was run using StrAuto v1.0 [43], allowing for parallel computation. To test the hypothesis of distinctive sub-populations within the major Brazilian biomes, a Bayesian clustering analysis was performed using the STRUCTURE admixture model assuming correlated allele frequencies for 10 replicates each of *K* = 1 through 7, with a ‘burn-in’ of 50,000 generations and a Markov chain Monte Carlo (MCMC) chain of 500,000 generations. CLUMPAK [44] was used to average runs and visualize STRUCTURE results. Principal components analysis (PCA) was conducted to test the hypothesis that the reduction of variables to principal components would lead to population separation based on SNP variation congruent with the populations from the Bayesian analysis. We performed PCA with the STRUCTURE file comparing different levels of population (biome, state, locality) in R (v. 3.6.0) using the *ade4* package v.1.7.13 [45] *via* the dudi.pca() function and visualized with *factoextra* package v.1.0.5 [46] fviz_pca_ind() function. To partition the genetic variation into clusters, and confirm the optimal cluster number, we employed discriminant analysis of principal components (DAPC) [47] with the R package *adegenet* v.2.1.1 [48]. A hierarchical analysis of molecular variance (AMOVA), with individuals grouped by locality within states, was calculated using the poppr.amova function in the R package *poppr* v.2.8.3 [49].

#### Life history trait analysis

All statistical analyses were conducted in R (v. 3.6.0) (Additional file 3: Dataset S2). A generalized linear model (GLM) was used to compare the effects of population (localities within state and families within localities) and temperature on life history traits. Genetic variation (populations or families), phenotypic plasticity (temperature levels) and genetic variation for plasticity (population/family-by-temperature interactions) were assessed for larval development, adult lifespan, and wing length with ANOVA (Type II). Comparison of localities within state was conducted on all states except for Rio de Janeiro because there was only one locality in that state (Fig. 1, Table 1). The Kaplan-Meier estimate of survival (time between larvae hatch and adult death) of individual families within each locality by temperature was visualized with the *survival* v.2.44.1.1 [50] and *survminer* v.0.4.3 [51] R packages.

#### Estimators of population differentiation: F_ST_ and P_ST_

Trait-based data can be used to estimate the amount of genetic variance among populations (*P*_ST_), which we expect to be comparable to calculated *F*_ST_. In order to compare population genetic structure results from the sequenced *P* generation (field-collected females) and their laboratory-reared F_1_ progeny, *F*_ST_ and *P*_ST_ values were calculated, respectively. Pairwise *F*_ST_ values by locality (for the 7 localities tested in the present study) were calculated with the *populations* program from STACKS [40] using the sequenced 93 field *Ny. darlingi*. Life history data of the reared progeny were used to estimate *P*_ST_, a phenotype-based analog for *F*_ST_ that measures the amount of genetic variation among populations relative to the total genetic variation [52], assuming that the proportion of phenotypic variance due to genetic effects is equivalent between and within populations. Pairwise *P*_ST_ values by locality for each life history trait were calculated with the *Pstat* R package v.1.2 [53]. The ratio of *P*_ST_ to *F*_ST_ values is a useful proxy for estimating the strength of selection [54] on phenotypic traits. As *F*_ST_ is typically estimated from neutral loci, deviations of *P*_ST_ from *F*_ST_ can lead to inferences of selection: if *P*_ST_ > *F*_ST_, directional selection can be inferred, conversely, if *P*_ST_ < *F*_ST_, stabilizing selection is indicated.

## Results

### Evidence of population genetic structure by major biome

**Fig. 2:**
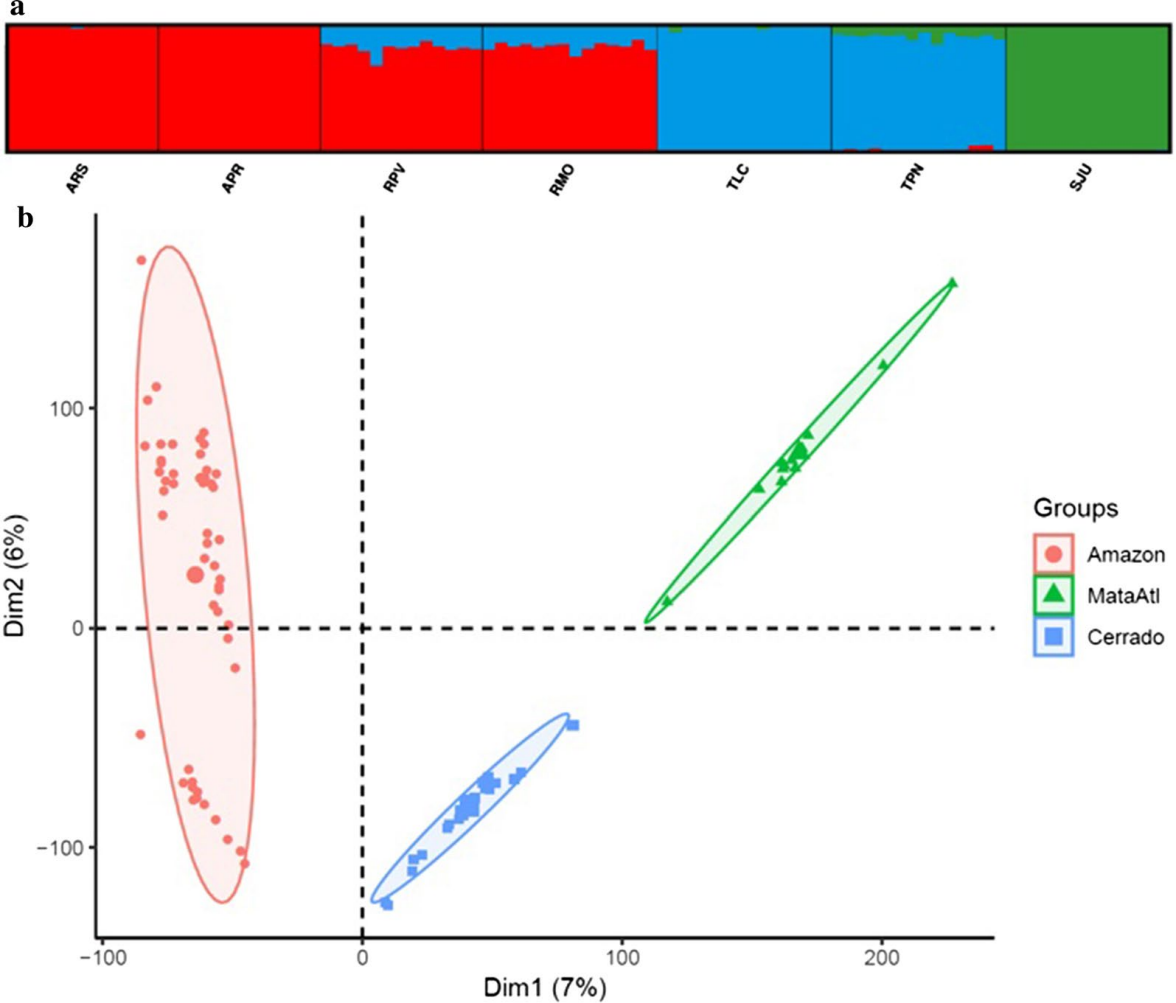
**a** STRUCTURE plot of 93 field-collected *Ny. darlingi* reveals clustering by major biome (*K* = 3). **b** Principal components analysis (PCA) by biome

There was an average of 3,891,842 (range: 359,767–6,636,895) sequences, or reads, per individual (n = 93) after quality filtering. The average number of reads per stack (or unique groups of matched reads) was 3,002,165 (range: 228,591–5,437,712) with an average number of 100,369 (range: 23,754–232,583) stacks per individual. The final SNP dataset included one biallelic SNP from each locus genotyped in at least 40% of the 93 individuals, for a total of 33,759 loci. The average coverage depth was 49X at each locus. Multiple values for *K* (*K* = 1–7) clusters were examined from STRUCTURE and STRUCTURE Harvester analyses (Additional file 4: Figure S1, Additional file 5: Figure S2). There was a dramatic dip in Δ*K* at *K* = 3 and the highest probability of *K* was at *K* = 3 (Additional file 4: Figure S1). The Bayesian information criterion (BIC) of the *K*-means clustering algorithm implemented in *adegenet* [47] in preparation for DAPC indicated *K* = 3 was the optimal number of clusters (Additional file 4: Figure S1b). Small amounts of admixture within the southern Amazonia biome (Rondônia state) samples were detected (Fig. 2a). Both the STRUCTURE (Fig. 2a, Additional file 5: Figure S2) and PCA (Fig. 2b) analyses identified three major clusters corresponding to the biome classifications.

### Variation of life history traits between localities within states

Increasing temperature reduced larval development time, adult lifespan, and wing length within all states (Additional file 1: Table S2, Additional files 6–8: Figures S3-S5). There were significant genetic differences between localities within Amazonas (*F*_(1, 968)_ = 52.0, *P* < 0.0001), Rondônia (*F*_(1, 1049)_ = 15.3, *P* < 0.0001) and Tocantins (*F*_(1, 332)_ = 6.7, *P* = 0.01) states for larval development time (Additional file 6: Figure S3), and only within Tocantins for adult lifespan (*F*_(1, 332)_ = 4.57, *P* = 0.03) (Additional file 7: Figure S4) and wing length (*F*_(1, 320)_ = 32.9, *P* < 0.0001) (Additional file 8: Figure S5).

The two localities within Amazonas State had significantly different larval development time at 20 °C (*t*_(968)_ = 3.77, *P* < 0.0001), whereas the two localities in Rondônia had significantly different larval development time at 20 °C (*t*_(1049)_ = 5.23, *P* < 0.0001) and 28 °C (*t*_(1049)_ = − 3.41, *P* < 0.0001). Only the localities in Amazonas State had significantly different adult lifespan (*t*_(968)_ = −  2.05, *P* = 0.04) and wing length (*t*_(940)_ = 2.44, *P* < 0.0001) at 24 °C. In contrast, localities in Tocantins had significantly different wing lengths at 20 °C (*t*_(320)_ = 2.44, *P* = 0.02) (Additional file 1: Table S2).

### Within-population genetic variation for traits and their plastic responses

**Fig. 3:**
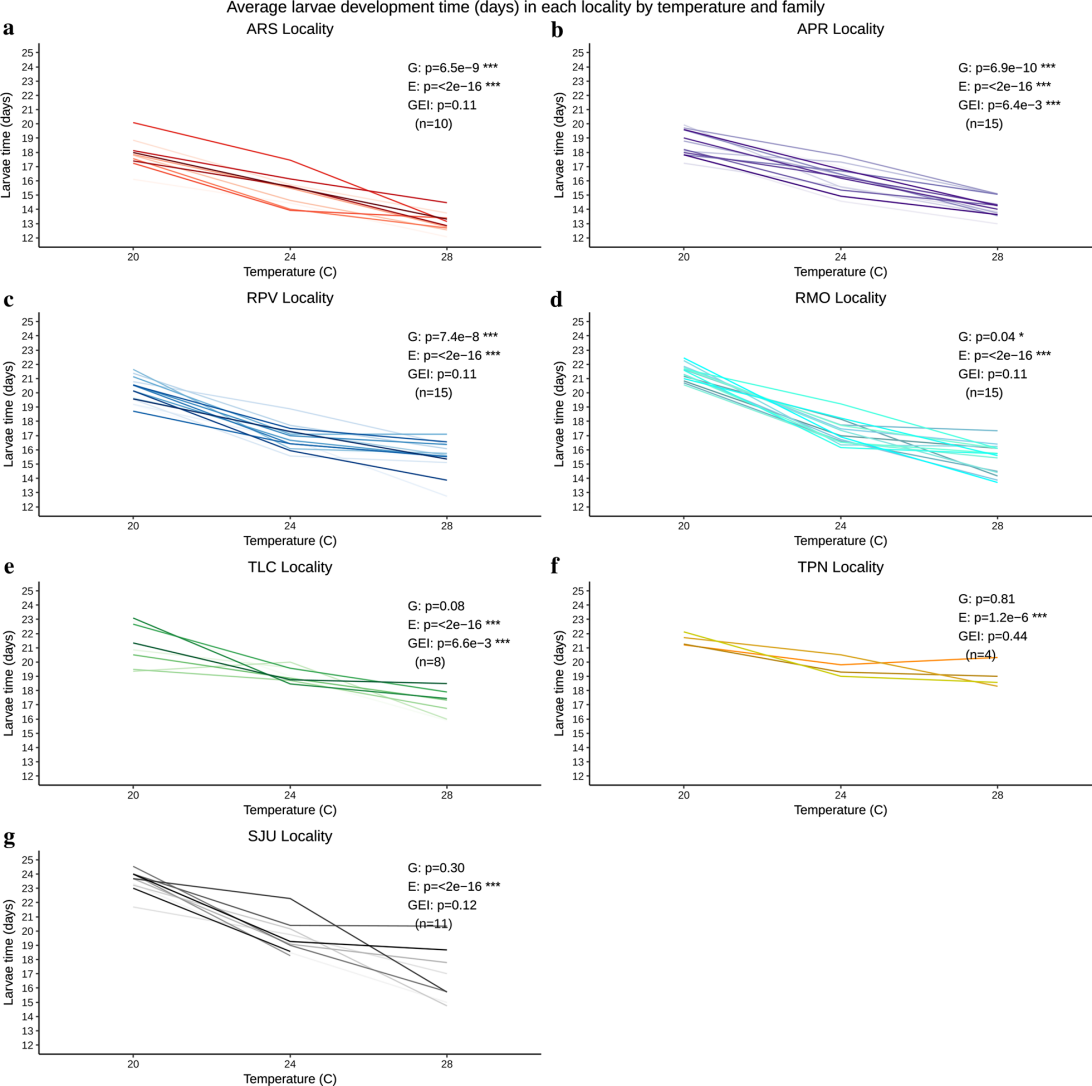
Average larval development time (days) of each family (uniquely colored line) by locality and temperature treatment (20, 24 and 28 °C) (**a**–**g**). ANOVA results in each panel: G, genetic variation (family); E, phenotypic variation (temperature); GEI, genotype-by-environment interaction (family × temperature); *P < 0.05, **P < 0.01, ***P < 0.001; n, number of families. *Abbreviations*: ARS, Ramal Novo Horizonte; APR, Manaus-Brasilierinho; RPV, Porto Velho; RMO, Machadainho D’Oeste; TLC, Lagoa da Confusão; TPN, Porto Nacional; SJU, Lake Juturnaiba

**Fig. 4:**
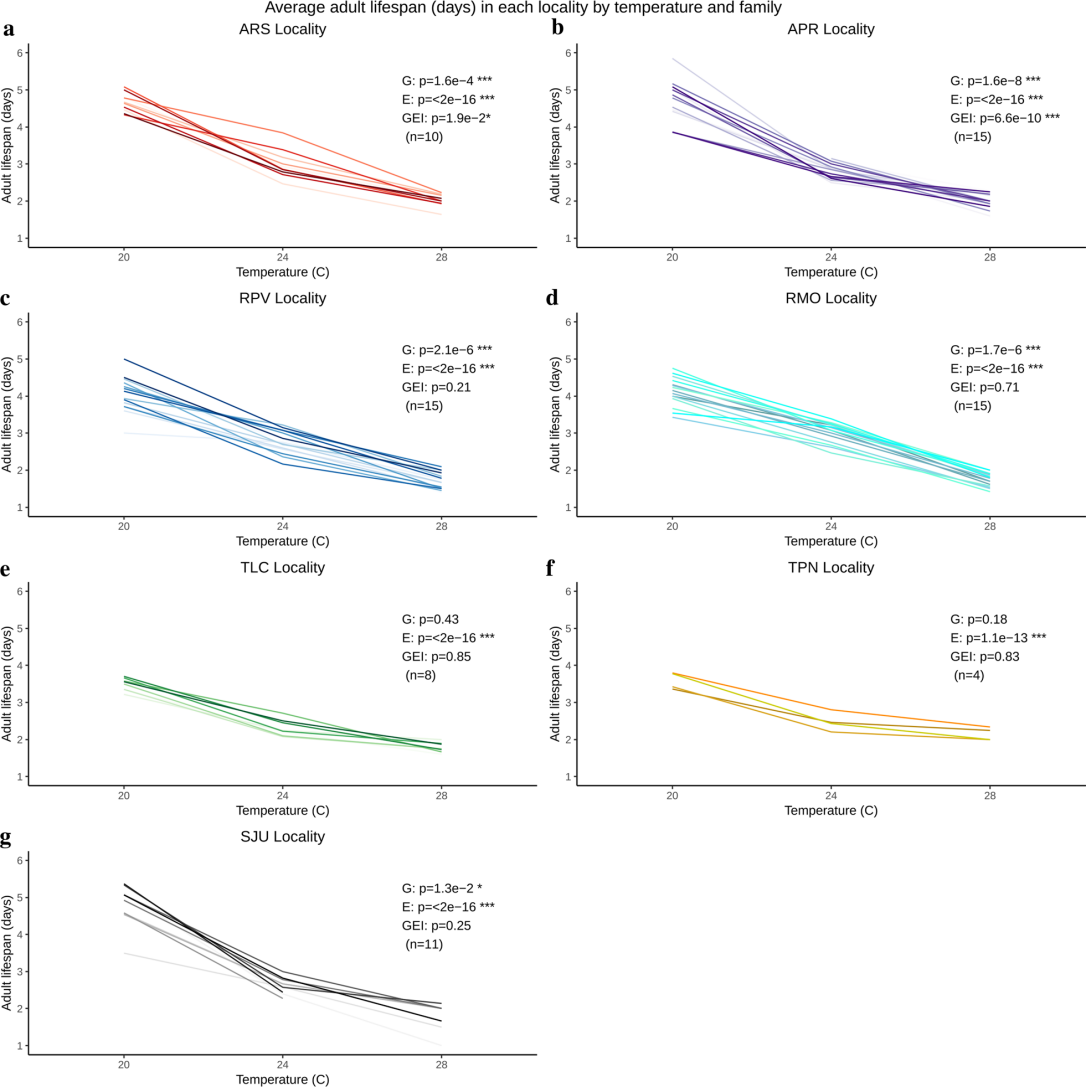
Average adult lifespan (days) of each family (uniquely colored line) by locality and temperature treatment (20, 24 and 28 °C) (**a**–**g**). ANOVA results in each panel: G, genetic variation (family); E, phenotypic variation (temperature); GEI, genotype-by-environment interaction (family × temperature); * *P* < 0.05, ** *P* < 0.01, *** *P* < 0.001; n, number of families. *Abbreviations*: ARS, Ramal Novo Horizonte; APR, Manaus-Brasilierinho; RPV, Porto Velho; RMO, Machadainho D’Oeste; TLC, Lagoa da Confusão; TPN, Porto Nacional; SJU, Lake Juturnaiba

**Fig. 5:**
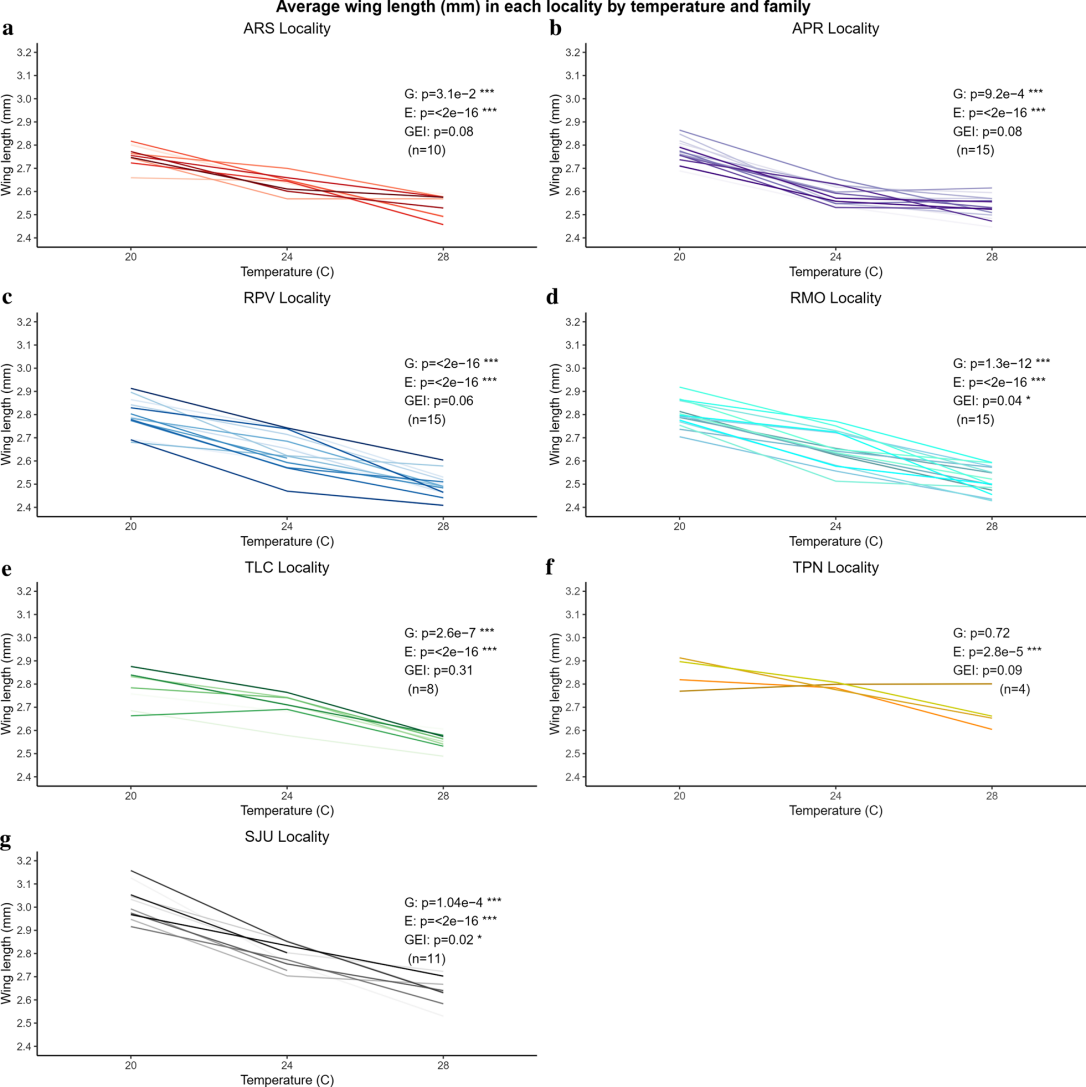
Average wing length (mm) of each family (uniquely colored line) by locality and temperature treatment (20, 24 and 28 °C) (**a**–**g**). ANOVA results in each panel: G, genetic variation (family); E, phenotypic variation (temperature); GEI, genotype-by-environment interaction (family × temperature); * *P* < 0.05, ** *P* < 0.01, *** *P* < 0.001; n, number of families. *Abbreviations*: ARS, Ramal Novo Horizonte; APR, Manaus-Brasilierinho; RPV, Porto Velho; RMO, Machadainho D’Oeste; TLC, Lagoa da Confusão; TPN, Porto Nacional; SJU, Lake Juturnaiba

There was significant genetic variation among families within populations for larval development time (ARS: *F*_(9, 369)_ = 6.71, *P* < 0.0001; APR: *F*_(14, 530)_ = 5.48, *P* < 0.0001; RPV: *F*_(14, 461)_ = 4.61, *P* < 0.0001; RMO: *F*_(14, 504)_ = 1.77, *P* = 0.04), adult lifespan (ARS: *F*_(9, 369)_ = 3.74, *P* < 0.0001; APR: *F*_(14, 530)_ = 4.88, *P* < 0.0001; RPV: *F*_(14, 461)_ = 3.94, *P* < 0.0001; RMO: *F*_(14, 504)_ = 3.96, *P* < 0.0001) and wing length (ARS: *F*_(9, 362)_ = 2.07, *P* = 0.03; APR: *F*_(14, 509)_ = 2.66, *P* < 0.0001; RPV: *F*_(14, 449)_ = 9.03, *P* < 0.0001; RMO: *F*_(14, 490)_ = 6.72, *P* < 0.0001) for both localities of Amazonas and Rondônia states (Figs. 3, 4, 5), and for adult lifespan (SJU: *F*_(10, 254)_ = 2.30, *P* = 0.01) and wing length (SJU: *F*_(10, 247)_ = 3.75, *P* < 0.0001) in the southern high latitude population. Populations from Tocantins showed little genetic variation among families, with the exception of wing length for TLC (*F*_(7, 214)_ = 6.82, *P* < 0.0001).

All traits from all populations showed significant responses to temperature - increasing temperature reduced larval development time, adult lifespan, and wing length in all localities (Figs. 3, 4, 5, Additional file 1: Table S2). The genotype-by-environment term was significant (or nearly so) for all three traits in multiple populations, indicating significant genetic variation among families for the response to temperature (different slopes among families; Figs. 3, 4, 5). Median survival times (larval hatch to adult death) of families within each locality were highest at 20 °C and decreased with increasing temperature. Family survival was significantly different within each locality at each temperature (Additional file 9: Figure S6).

### Estimators of population differentiation: AMOVA, *F*_ST_ and *P*_ST_

The hierarchical AMOVA (Table 2) revealed highly significant levels of variation at each level (*P* < 0.001), and the genetic variation was mainly accounted for within individuals (71.8%), followed by among individuals (19.1%) and between states (9.1%). Pairwise *F*_ST_ between localities of the field collected females (*P* generation) ranged from 0.045 to 0.183, with the lowest *F*_ST_ values between paired sites within the same state, as expected (Table 3). The calculated pairwise *P*_ST_ values across all lab-reared progeny (F_1_ generation) by locality were generally greater than *F*_ST_ for larval development (range: 0.660–0.995) (Table 4), adult lifespan (range: 0.0004–0.972) and wing length (range: 0.211–0.994) (Table 5), with few exceptions. The lowest pairwise *P*_ST_ values for these three traits were between localities within the same state (Tables 4, 5). Comparison of *P*_ST_ values for progeny life history traits with parental *F*_ST_ values reveals that *P*_ST_ values are nearly all substantially greater, an indication that directional selection is responsible for some of the genetic differentiation of life history traits among regions.

**Table 2:** Analysis of molecular variance (AMOVA), with individual *Ny. darlingi* (*n* = 93) nested within localities within states

Source of variation	*df*	Sum of squares	Variance components	Percentage of variation	*P*-value^a^
Among states	3	227031.9	1292.19	9.063368	0.001
Among individuals within state	3	139581.8	2724.092	19.106667	0.001
Within individuals	86	880726.2	10241.002	71.829965	0.001
Total	92	1247339.8	14257.284	100	

^a^Based on 999 Monte-Carlo permutation tests

*Abbreviation*: *df*, degrees of freedom

**Table 3:** Pairwise *F*_ST_ between localities for field collected (P) generation

	APR	RPV	RMO	TLC	TPN	SJU
ARS	**0.046**	0.067	0.070	0.106	0.100	0.183
APR		0.062	0.066	0.103	0.096	0.172
RPV			**0.045**	0.089	0.081	0.149
RMO				0.092	0.081	0.149
TLC					**0.061**	0.132
TPN						0.120

*Note*: Within-state comparisons are indicated in bold

*Abbreviations*: ARS, Ramal Novo Horizonte; APR, Manaus-Brasilierinho; RPV, Porto Velho; RMO, Machadainho D’Oeste; TLC, Lagoa da Confusão; TPN, Porto Nacional; SJU, Lake Juturnaiba

**Table 4:** Pairwise *P*_ST_ between localities of F_1_ generation for larval development time

	ARS	APR	RPV	RMO	TLC	TPN	SJU
ARS		**0.935**	0.986	0.988	0.993	0.991	0.996
APR			0.970	0.980	0.990	0.988	0.995
RPV				**0.757**	0.964	0.966	0.989
RMO					0.911	0.928	0.982
TLC						**0.704**	0.933
TPN							0.660
SJU							

*Note*: Pairwise *F*_ST_ values calculated between *populations* of field-collected *Ny. darlingi* by locality. Within-state comparisons are indicated in bold

*Abbreviations*: ARS, Ramal Novo Horizonte; APR, Manaus-Brasilierinho; RPV, Porto Velho; RMO, Machadainho D’Oeste; TLC, Lagoa da Confusão; TPN, Porto Nacional; SJU, Lake Juturnaiba

**Table5:** Pairwise *P*_ST_ for adult lifespan and wing length of laboratory reared F_1_ generation

	ARS	APR	RPV	RMO	TLC	TPN	SJU
ARS	–	**0.0004**	0.832	0.771	0.959	0.861	0.762
APR	**0.394**	–	0.843	0.787	0.956	0.840	0.764
RPV	0.517	0.788	–	**0.184**	0.901	0.589	0.936
RMO	0.722	0.871	**0.211**	–	0.923	0.687	0.926
TLC	0.870	0.919	0.693	0.565	–	**0.472**	0.972
TPN	0.965	0.971	0.942	0.941	**0.919**	–	0.917
SJU	0.993	0.994	0.991	0.991	0.986	0.887	–

*Notes*: Pairwise *P*_ST_ values calculated between *populations* for larvae development time (days), adult lifespan (days) and wing length (mm)

Within-state comparisons are indicated in bold

*Abbreviations*: ARS, Ramal Novo Horizonte; APR, Manaus-Brasilierinho; RPV, Porto Velho; RMO, Machadainho D’Oeste; TLC, Lagoa da Confusão; TPN, Porto Nacional; SJU, Lake Juturnaiba

## Discussion

Using a representative sample of fine-scale SNP data, and an extensive common-garden experiment, we found strong evidence of among-population genetic differentiation as well as within-population variation in plasticity in major life history traits. In particular, our SNP analysis found population differentiation according to major biome designation rather than at a local scale, similar to previous findings [37]. We also found evidence of variation in plasticity in life history traits at local scales. The genetic structure of *Ny. darlingi* populations as well as the local-level variation for plasticity of this vector has major implications for the future of malaria elimination in South America. A recent report from the World Health Organization highlighted the importance of novel and local approaches as vital for malaria elimination [55] and our findings indicate that the variation at the local level could enable some populations to potentially tolerate changing temperature (i.e. Amazonia biome) and for potential increased local transmission in southern populations (i.e. Rio de Janeiro).

In partial support of previous findings of differentiation according to biome and physical barriers [37], populations analyzed in the present study clustered by major biome. A new finding was the detection of low admixture between the two states at the same latitude (Rondônia, Tocantins) (Fig. 2). Our study revealed geographical divisions, with pairwise *F*_ST_ between localities (34–120 km apart) lowest within (range: 0.046–0.070) compared to between biomes (range: 0.081–0.183) (Table 3) indicating weaker genetic differentiation at smaller geographical scales. These findings suggest that biome boundaries may represent strong barriers to gene flow in *Ny. darlingi*. There is limited evidence from SNP analysis of significant microgeographical genetic differentiation of *Ny. darlingi* from western Amazonian Brazil related to varying levels of deforestation among municipalities (that ranged from 60–1600 km apart); one study detected significant differentiation when comparing an older, highly deforested agricultural settlement 60 km from a newly settled one that retained high forest cover levels [38], and another detected low and non-significant variation when comparing multiple deforestation levels among several Brazilian Amazon settlements [42].

A study of the closely related species *An. gambiae* (*s.s.*) and *An. coluzzii*, collected from 15 sites across Africa and tested with over 50 million SNPs, revealed clustering by geographical region rather than species, and as predicted, lower *F*_ST_ values within rather than between biomes [56]. Population structure of *Anopheles* species that have been analyzed is strongly affected by geographical division; such demarcations may break down in the future as the integrity of biomes is eroded by deforestation and climate change. Specifically, in South America, under a high CO_2_ emission model, there could be substantial reduction (3%) in tropical forest area in South America in the next 10 years and up to 18% by 2100 [57]. As *Ny. darlingi* is primarily associated with forested areas, its range [58] and population structure will likely be altered. Variation at the individual level was high in this study (72%) and suggests potential for adaptation.

Our study extends previous findings of regional variation in life history traits of *Ny. darlingi* [7] to the detection of significant genetic variation within localities. Significant genetic variation between families was found consistently within populations within the Amazonia biome (larval development time, adult lifespan and adult body size) and families from the Mata Atlântica exhibited significant genetic variation for adult lifespan and body size. These populations (Amazonas and Mata Atlântica) may have greater adaptive potential to increase their resistance to changing temperature given their responses in the laboratory experiment. *P*_ST_ values [52] of localities within each state were the lowest for all three life history traits (larval development time, adult lifespan and wing length), as expected. Because *P*_ST_ values are nearly uniformly substantially greater than the parental *F*_ST_ values, we infer that there is directional selection driving genetic differentiation of life history traits among regions. Combined with our evidence that there is standing genetic variation for performance at different temperatures within populations, future selection could favor phenotypes that tolerate increased temperatures.

The environment substantially influences mosquito vector traits. In our study, increased temperature reduced larval development time in all populations with the magnitude of reduction in adult lifespan and body size dependent on population. The dramatic differences in larval development time however, were not a clear predictor of adult longevity due to differences between populations. Our data show that the relationship between larval conditions and adult traits is not linear but rather complex. Temperature of larval and adult environments had significant effects on *An. gambiae* (*s.s.*) development: increased larval rearing temperature (23–31 °C) resulted in smaller larvae and adults whereas increased adult temperatures reduced the proportion of egg hatch [30]. The effects of temperature can reduce mosquito population size over time, with smaller individuals laying fewer eggs. On the other hand, smaller increases in temperature can increase mosquito population size, with a field study of *An. gambiae* (*s.s.*) in Kenya revealing greater fecundity and vectorial capacity of mosquitoes placed in homes that were 0.7–1.2 °C warmer compared with control homes [59].

Vector control interventions need to consider variation in life history traits [7], behaviors [5], and habitats [21]. At a high nutrition diet, low temperature treatment *Anopheles* were found to be larger and more likely to survive exposure to a LC_50_ dose of permethrin [60]. Data from our study suggest that larger doses of permethrin would be required in southern compared to northern populations of *Ny. darlingi*. Interventions such as long-lasting insecticidal nets (LLINs) are highly effective and target adult mosquitoes that are mainly endophagic and endophilic. The biting behavior of *Ny. darlingi* is variable [41, 61], compromising the efficacy of IRS or LLINs. Field studies of *Ny. darlingi* reveal endophagy and exophagy at different times throughout the night [5], and there is no evidence for a genetic basis of these behaviors [41]. Ivermectin treatment of cattle was shown to reduce *An. arabiensis* fecundity by nearly 60% after deployment of LLINs compared to LLINs alone, supporting the use of combination interventions to help achieve population elimination [62].

The plasticity of *Ny. darlingi*, including biting behavior [5, 41], host [12, 63] and breeding site [19–21] preferences, coupled with the potential of families within certain populations to withstand changing environments, help explain its status as the major malaria vector in South America. The present study contributes to the growing body of evidence of high levels of plasticity in *Ny. darlingi*, and significantly, presents evidence for genetic variation in plasticity *within* populations.

A potential study limitation was that we were unable to collect *Ny. darlingi* from a second locality in Rio de Janeiro although we had previous evidence of its presence [37]. This limited the comparison of life history trait responses from paired localities within Rio de Janeiro State and reduced our ability to adequately test population structure within this biome. While the *F*_ST_ values were calculated from field collected mosquitoes and *P*_ST_ values from laboratory-reared progeny, it is unlikely that there would accrue significant genetic variation between parent and offspring in one generation. To date, *Ny. darlingi* has not been tested for polyandry, although *An. gambiae* exhibits low polyandry (12%) [64] whereas nearly 25% of *An. arabiensis* females had been multiply inseminated [65]. We treated individuals within families as full-siblings assuming that polyandry did not contribute significantly to the observed variation between families.

The study design of our laboratory experiment to observe life history traits at a constant temperature throughout the mosquito life cycle was somewhat limited by space and resources. The temperatures in this experiment were chosen to avoid extremes that can lead to excessive mortality that would limit comparisons [30], and reflect averages at specific latitudes; they may not reflect specific microclimates for each locality. The temperature range (8 °C) we used may not reflect actual temperatures projected for Brazil under climate change [16, 66, 67]. Research with established laboratory colonies has also shown that fluctuating temperatures may more accurately reflect the natural environment, and affect life history traits differently compared to constant temperatures [68].

Our treatment of adult mosquitoes (providing only water, no food) deviates from the natural adult environment which involves sugar feeding and potential blood meals for females as well as sugar-feeding for males. On the other hand, the average adult longevity in our study was 3.09 days compared to field data of daily survival rates that detected between 3.73 and 23.9 days for adult females from two Peruvian sites [12]. Our research did not investigate variation in biting behavior, fecundity and susceptibility to *Plasmodium* that can be affected by temperature and *Ny. darlingi* population specificity. The establishment and maintenance of a laboratory colony in Peru [69] and Brazil [70] as well as successful *Plasmodium* infection of colony mosquitoes [71] would facilitate investigations of this variation over generations and between populations.

## Conclusions

This study identified the population structure and degree of genetic variation and phenotypic plasticity of *Ny. darlingi* in Brazil. The genomic signatures indicate that genetic divergence occurs at the level of biomes, with phenotypic traits varying more than molecular markers indicating a role for natural selection *via* climate or vegetation structure in driving differentiation. A key result is our finding that there is genetic variation for both life history traits and their plastic responses within populations to temperature, indicating future adaptive capacity to changes in temperature. Future research that further quantifies the effects of environment and population on life history traits relevant to transmission will be vital for predicting variation in transmission potential and informing modeling efforts.

## Supplementary information

**Additional file 1: Table S1.** Number of larvae and families from each locality at each temperature. **Table S2.** Generalized linear model results for larval development, adult lifespan, and wing length. **Table S3.** Supplemental collection site data.

**Additional file 2: Dataset S1** STRUCTURE file used for STRUCTURE, PCA, DAPC and *F*_ST_ analyses, with genotypes for 93 individuals at 33,759 loci.

**Additional file 3: Dataset S2.** Life history data used for ANOVA, *P*_ST_, GLM and Kaplan–Meier analyses.

**Additional file 4: Figure S1.** Estimation of the number of clusters in SNP dataset using STRUCTURE and Discriminant Analysis of Principal Components (DAPC). **a** STRUCTURE *ΔK* using Evanno method (left) and probability by *K* (right) [44] for *K* = 1–7. **b** Bayesian information criterion (BIC) for 1 to 20 clusters using *K* means clustering preparation for DAPC.

**Additional file 5: Figure S2.** STRUCTURE plots for *K* = 1–7.

**Additional file 6: Figure S3.** Average larval development time of localities within each state. Standard error bars and ANOVA results in each panel: G, genetic variation (locality); E, phenotypic variation (temperature); GEI, genotype-by-environment interaction (locality × temperature); *P < 0.05, **P < 0.01, ***P < 0.001.

**Additional file 7: Figure S4.** Average adult lifespan of localities within each state. Standard error bars and ANOVA results in each panel: G, genetic variation (locality); E, phenotypic variation (temperature); GEI, genotype-by-environment interaction (locality × temperature); *P < 0.05, **P < 0.01, ***P < 0.001.

**Additional file 8: Figure S5.** Average wing length of localities within each state. Standard error bars and ANOVA results in each panel: G, genetic variation (locality); E, phenotypic variation (temperature); GEI, genotype-by-environment interaction (locality × temperature); *P < 0.05, **P < 0.01, ***P < 0.001.

**Additional file 9: Figure S6.** Kaplan-Meier survival curve of families within locality. Each family is uniquely color coded and consistent with all other figures’ color scheme for family.

## Data Availability

Raw Illumina sequences were deposited in the NCBI Sequence Read Archive (SRA; BioProject ID PRJNA576174). All other relevant data are within the manuscript and its additional files.

## Abbreviations

AMOVA: analysis of molecular variance
ANOVA: analysis of variance
APR: Manaus-Brasilierinho, Amazonas State, Brazil
ARS: Ramal Novo Horizonte, Amazonas State, Brazil
BIC: Bayesian information criterion
DAPC: discriminant analysis of principal components
F_1_: offspring generation 1
*F*_ST_: fixation index
GLM: generalized linear model
LLINs: long-lasting insecticidal nets
MCMC: Markov chain Monte Carlo
nextRAD: nextera-tagmented, reductively-amplified DNA
P: parent generation
PCA: principle components analysis
*P*_ST_: degree of phenotypic differentiation
R_0_: basic reproductive rate
RMO: Machadinho dʼOeste, Rondonia State, Brazil
RPV: Porto Velho, Rondonia State, Brazil
SJU: Lake Juturnaiba, Rio de Janeiro State, Brazil
SNP: single nucleotide polymorphism
TLC: Lagoa da Confusão, Tocantins State, Brazil
TPN: Porto Nacional, Tocantins State, Brazil
