# Supplementary figures and images for: Evidence for family-level variation of phenotypic traits in response to temperature of Brazilian Nyssorhynchus darlingi
Source: Parasit Vectors. 2020 Feb 10;13:55. doi: 10.1186/s13071-020-3924-7 (PMC7011564; doi:10.1186/s13071-020-3924-7)

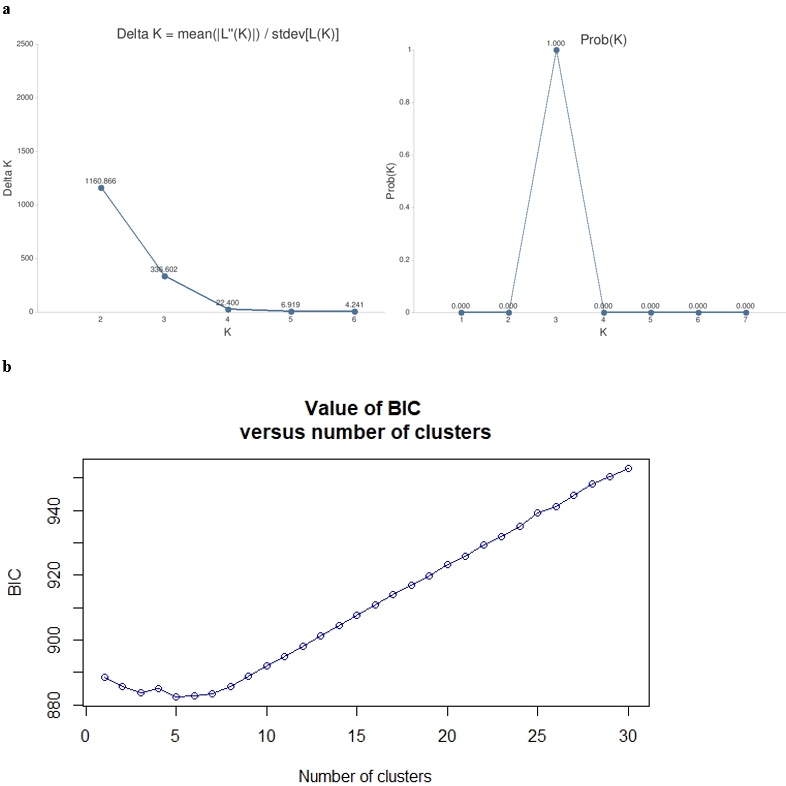

Supplement: Supplementary file 4 — Additional file 4: Figure S1. Estimation of the number of clusters in SNP dataset using STRUCTURE and Discriminant Analysis of Principal Components (DAPC). a STRUCTURE ΔK using Evanno method (left) and probability by K (right) [44] for K = 1–7. b Bayesian information criterion (BIC) for 1 to 20 clusters using K means clustering preparation for DAPC. [file 13071_2020_3924_MOESM4_ESM.jpg]

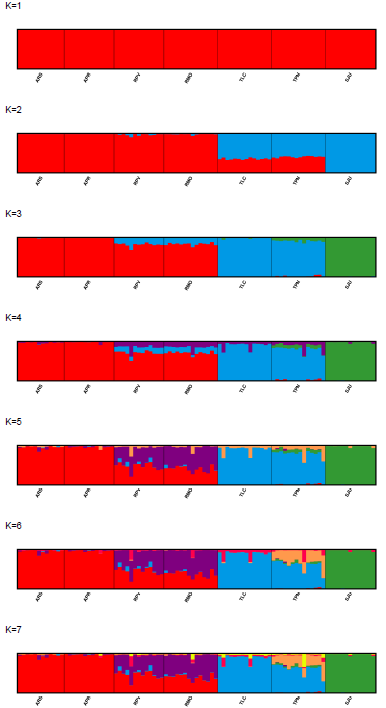

Supplement: Supplementary file 5 — Additional file 5: Figure S2. STRUCTURE plots for K = 1–7. [file 13071_2020_3924_MOESM5_ESM.png]

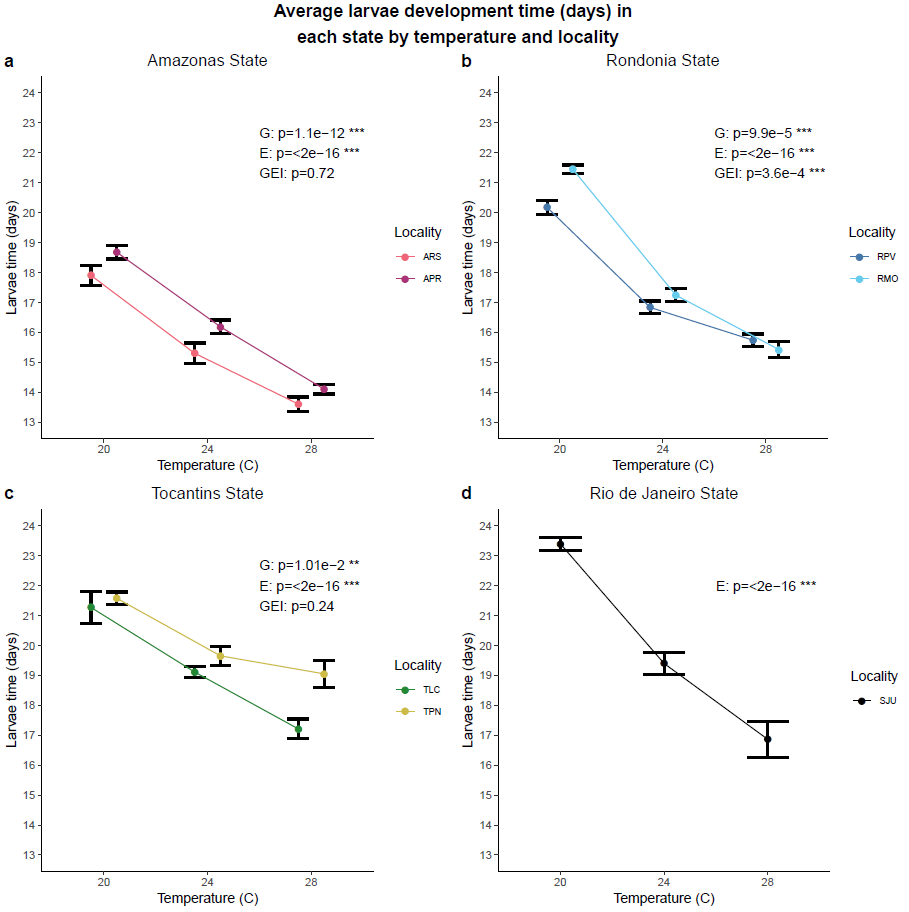

Supplement: Supplementary file 6 — Additional file 6: Figure S3. Average larval development time of localities within each state. Standard error bars and ANOVA results in each panel: G, genetic variation (locality); E, phenotypic variation (temperature); GEI, genotype-by-environment interaction (locality × temperature); *P < 0.05, **P < 0.01, ***P < 0.001. [file 13071_2020_3924_MOESM6_ESM.png]

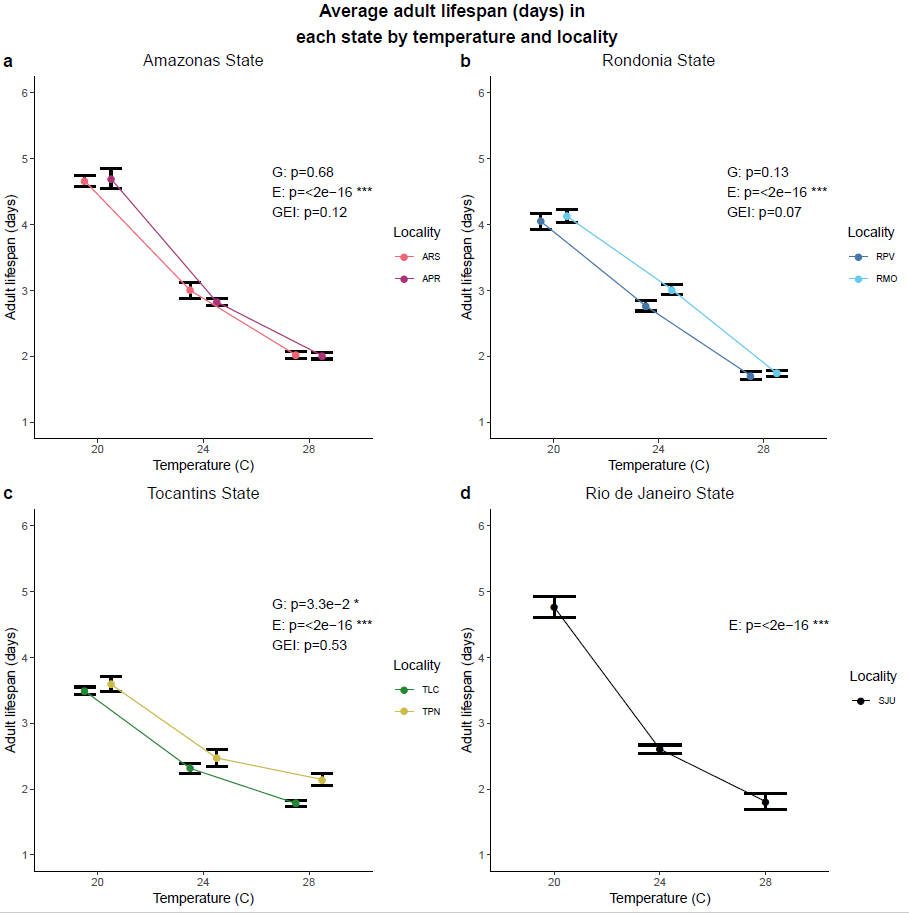

Supplement: Supplementary file 7 — Additional file 7: Figure S4. Average adult lifespan of localities within each state. Standard error bars and ANOVA results in each panel: G, genetic variation (locality); E, phenotypic variation (temperature); GEI, genotype-by-environment interaction (locality × temperature); *P < 0.05, **P < 0.01, ***P < 0.001. [file 13071_2020_3924_MOESM7_ESM.png]

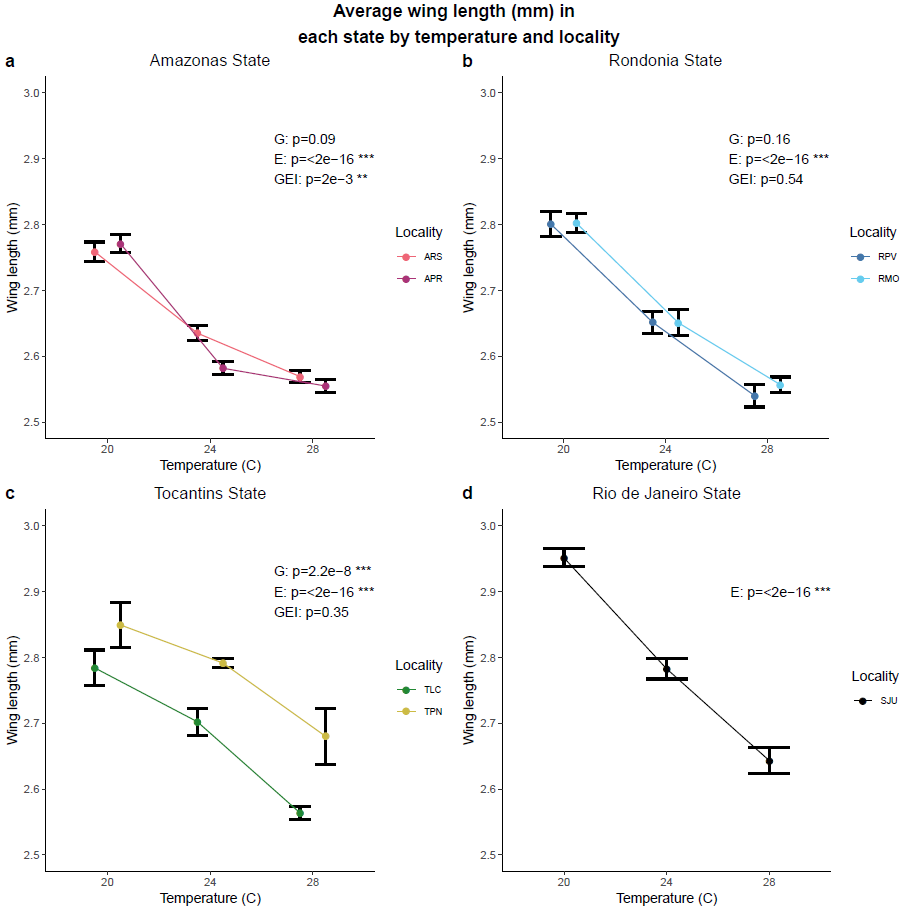

Supplement: Supplementary file 8 — Additional file 8: Figure S5. Average wing length of localities within each state. Standard error bars and ANOVA results in each panel: G, genetic variation (locality); E, phenotypic variation (temperature); GEI, genotype-by-environment interaction (locality × temperature); *P < 0.05, **P < 0.01, ***P < 0.001. [file 13071_2020_3924_MOESM8_ESM.png]

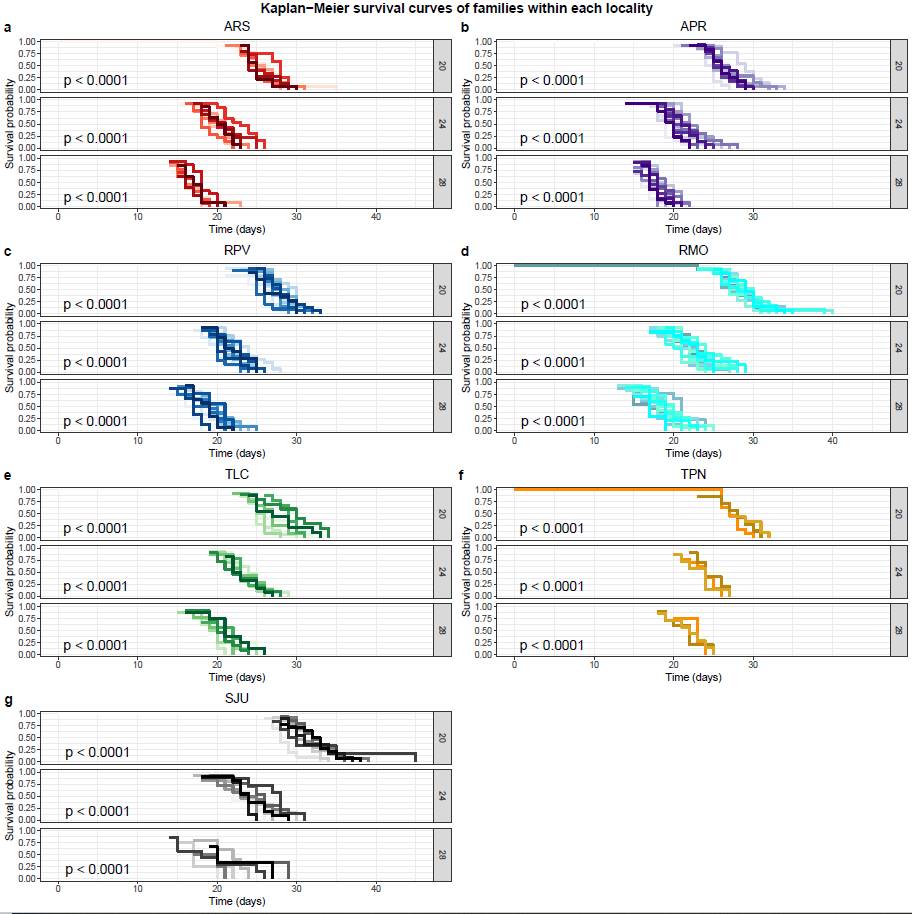

Supplement: Supplementary file 9 — Additional file 9: Figure S6. Kaplan-Meier survival curve of families within locality. Each family is uniquely color coded and consistent with all other figures’ color scheme for family. [file 13071_2020_3924_MOESM9_ESM.png]
